# Supplementary material for: Improving micromorphological analysis with CNN-based segmentation of flint/obsidian, bone and charcoal
Source: PLoS One. 2026 Jan 20;21(1):e0340353. doi: 10.1371/journal.pone.0340353 (PMC12818656; doi:10.1371/journal.pone.0340353)
Supplement: Attachment — Submitted filename: rebuttal.pdf [file pone.0340353.s001.pdf]

The authors greatly appreciate the provided suggestions, which have been addressed as described below:

- The style requirements for PLOS ONE have been applied to the manuscript, including authors information, figure citations, section title capitalisation, and file naming.
- Additional information regarding the permits required for our study has been included in lines 107–109 of the manuscript
- A code and data availability statement has been included at the end of the manuscript. Legal restrictions over the experimental dataset have been lifted and it is now freely available along with the code.
- The manuscript has been corrected to use British spelling and the abstract has been modified to expand all abbreviations, to avoid unnecessary architecture names, and to include representative values for bone and charcoal, minimal dataset context, and numeric values to substantiate the claim of high balanced accuracy for abundance classification. As recommended, ocurrences of “flint/obsidian” have been replaced with “flint (including obsidian)”.
- Keywords have been modified to avoid duplicating title phrases, as well as acronyms and slash forms.
- The *Results* and *Discussion* sections have been separated into distinct, substantive sections. As suggested, the newly added *Discussion* section includes an interpretation of performance differences between classes, typical failure modes and their causes, the influence of annotation quality and class imbalance, risks of data leakage (subsection “Class performance and methodological considerations” and further extended in subsection “Annotating difficulties and future directions”); the extent of generalisability, the limitations and potential biases of the study, and outline practical implications for archaeological micromorphology and directions for future work (subsection “Archaeological impact and applicability”).
- The conclusion section has been expanded to explicitly state the limitations and the bounds of generalisability of the presented study. As explained in the newly added “Data availabilty statement”, both the code and all experimental data are now publicly available. Key methodological risks have been acknowledged and the tone of the text has been adjusted to align with the evidence presented.
- Incorrect decimal separators and table captions have been fixed.

- To the best of our knowledge, all inconsistent spelling ("optimise", "penalise", "summarise"...) and typographical mistakes have been corrected.
- Specific information regarding image tagging duties has been added to the "Dataset" subsection of "Materials and methods".

Best regards,

Javier Hernández-Aceituno
