# Supplementary material for: Improving micromorphological analysis with CNN-based segmentation of flint/obsidian, bone and charcoal
Source: PLoS One. 2026 Jan 20;21(1):e0340353. doi: 10.1371/journal.pone.0340353 (PMC12818656; doi:10.1371/journal.pone.0340353)
Supplement: Attachment — Submitted filename: rebuttal_auresp_2.pdf [file pone.0340353.s002.pdf]

The authors greatly appreciate the provided suggestions, which have been addressed as described below:

Editor's comments:

– First, although you explain why flint and obsidian were merged into a single class, it would be helpful to state explicitly whether the model performs equally well on both materials and whether this merging was driven exclusively by sample size or also by their practical indistinguishability in PPL/XPL imagery. This brief clarification, which the reviewer also noted, will be useful for future users of the dataset.

The following phrase has been included in the first paragraph of Materials and Methods for clarification: "Although these materials are different mineralogically and can be distinguished using Crossed Polarized Light (XPL), their conchoideal type of fracture, their shape, size, and optical characteristics under Plane Polarized Light (PPL) are similar."

– Second, the manuscript currently describes the thin sections as originating from "different contexts, regions and time periods". For transparency and archaeological replicability, please provide one additional clarifying sentence indicating at least the broad geographic areas or approximate chronological ranges represented in the dataset. This does not require detailed provenance information, merely a minimal contextual indication.

The following sentence has been included for contextual indication: "These photomicrographs were obtained from [...] contexts where these materials are abundant. Chronologies and geographical areas include European and Near Eastern Middle Palaeolithic, Iberian Peninsula Neolithic and Ancient Canarian and experimental samples studied in Mallol et al. (2013)."

Reviewer's comments:

– The phrase "(...) flint (including obsidian)" suggests that obsidian is a subtype of flint. Please rephrase to clarify that they were clustered together for methodological reasons, not because of material taxonomy. Correct it throughout.

The text has been rephrased to "lithic fine-grained debitage (flint and obsidian)" and corrected throughout the whole manuscript.

– Moreover, are flint and obsidian truly indistinguishable in this approach? Is the model equally reliable with both, or could there be differences? Since not all Palaeolithic contexts contain obsidian, and many use other types of chert,

this limitation should be acknowledged.

The following sentence has been included in "Annotating difficulties and future directions" and "Conclusions" respectively: "the dataset will be expanded to include a greater number of these materials, along with additional fine-grained lithic raw materials such as chert and quartzite," and "Furthermore, several archaeological contexts use chert, quartzite or other fine and coarse-grained materials that are not represented in this study."

Introduction:

– Lines 14–17: add references.

References Angelucci (2003) and Huisman et al. (2014) were added to the paragraph.

– Lines 22–23: explain the statement and cite if not your own reasoning. Lines 24–25: this does not need a new paragraph.

The statement was merged with the next paragraph, where it is explained.

– Lines 35-51: this is the core of the introduction, but it is currently diffuse. Please rewrite to highlight clearly the aim, how, and significance of the study.

To enhance the clarity of the aim and objectives of the article, we propose to merge the two paragraphs into one: "To enhance the reliability of micromorphological data, it is essential to maximize objectivity and, where possible, introduce quantitative precision to support interpretation. With this in mind, the primary aim of our project is to develop a tool using image–based ML that facilitates the identification and quantification of some of the most common materials in Palaeolithic contexts and thin sections: bone (fresh, degraded, burnt and calcined), charcoal and fine-grained lithic debitage (flint and obsidian)."

– Lines 44-48: add references.

As suggested in the response above, these paragraphs have been removed.

– Lines 52-58: the tone is too didactic; I suggest trimming or rephrasing. The last paragraph of the introduction should state succinctly: what you propose, why it matters, and what novelty it brings to the field.

The paragraph has been removed. The introduction now finalizes with stating the aims of the article.

Related work

– As written, this section feels isolated. Consider integrating the discussion of ML applications into Introduction, Methods, or Discussion instead of leaving it standalone.

The paragraph has been moved to the introduction, before the "aims" paragraph.

– Lines 66-74 (Big Data vs Slow Data): this paragraph is disconnected. If you keep it, please explain why this distinction matters. Line 71 needs a reference.

The paragraph "Big Data vs Slow Data" has been removed, as well as lines 86-93 as they felt disconnected.

Materials and methods

– In Fig. 1 use the same terminology (Flint/Obsidian) as in the text.

The legend of figure 1 has been modified to match the terminology of the rest of the work.

– Table 2: unify the number format and apply a consistent rounding rule across rows.

All numeric data in table 2 have been unified to fixed format with two decimal digits.

– Line 183: no need to re-explain the acronym after the first mention; please correct throughout.

All redefinitions of the IoU acronym have been removed, except the first instance and within the abstract.

– Please provide more detail about the origin of contexts and the experimental samples: where do they come from, and how were they created?

The following sentence has been included for contextual indication, following the editor's comments: "These photomicrographs were obtained from [...] contexts where these materials are abundant. Chronologies and geographical areas include European and Near Eastern Middle Palaeolithic, Iberian Peninsula Neolithic and Ancient Canarian and experimental samples studied in Mallol et al. (2013)."

Results

– *Table 6: write Flint/obsidian or explain the abbreviation.*

An explanation of the abbreviation has been included in the caption of Table 6.

– *This section contains methodological notes and interpretive comments that should be moved to Methods and/or Discussion. The Results section should be reserved for data only.*

The second paragraph of the Results section has been partially removed, as its information was already discussed in the Materials and Methods section. Similarly, several paragraphs discussing the meaning of the results have been moved to the beginning of the Discussion section.

– *Lines 273-275: this partly duplicates the caption of Fig. 5. Please integrate or remove repetition.*

The redundant paragraph has been removed in favor of the figure caption.

Discussion

– *Lines 339-341: the phrasing is too promotional; please reword to avoid sensational tone.*

The sentence has been reworded as "The possibility of automating part of the identification and quantification process constitutes a practical improvement: it would reduce the time micromorphologists spend on manual annotation and counting, while still providing results that are consistent and dependable."

– *The archaeological impact would benefit from more concrete examples: how exactly would this tool improve interpretation of hearth features, stratigraphic complexity, or activity areas?*

The following paragraph was added: "This methodology would enhance the quantification of these elements in archaeological features. For instance, in the characterization of combustion structures, where charcoals are frequently found, or the identification of surface occupations and activity areas, characterized by high abundance of bones and fine-grained lithic debitage."

– *The limitations section should note that charcoal, in particular, requires more samples and more morphological variability to improve training.*

The paragraph was rephrased as follows: "At the same time, the dataset will be expanded to include a greater number of these materials, along with

additional fine-grained lithic raw materials such as chert and quartzite, as well as charcoal exhibiting greater morphological variability and bone fragments. Other materials expected to be incorporated into the dataset include pottery fragments, specific rock lithologies, and malacofauna, thereby broadening the applicability of the model."

Other comments

– In figure titles, you can avoid phrasing such as "Results of ...".

The captions of figures 2, 3 and 4 have been simplified by removing the redundant wording.

– Avoid one–sentence paragraphs throughout the text; merge with adjacent ones for smoother reading.

To improve readability, all one-sentence paragraphs have been merged into larger bodies, as marked by highlighted initial letters in the manuscript, or removed.

– A workflow figure (dataset → annotation → model training → results) would improve accessibility.

A workflow figure has been added at the end of the Materials and Methods section, as suggested.

– Standardise terminology: Flint/Obsidian throughout.

All occurences of the term have been standardised in the manuscript.

– Ensure all abbreviations are expanded at first use (IoU, PPL, XPL) and no need to expand them in the following occurrences (e.g., line 183).

All abbreviations have been fixed, expanding them only on their first appearance.

– Improve figure/table captions so they are more self-explanatory.

The captions of tables 6 and 7 have been reworded to be more self-explanatory.

Best regards,
Javier Hernández-Aceituno
